# Barriers, Attitudes and Clinical Approach of Lebanese Physicians Towards HPV Vaccination; A Cross- Sectional Study

**DOI:** 10.31557/APJCP.2019.20.10.3181

**Published:** 2019

**Authors:** Joseph Abi Jaoude, Halim Saad, Loulwa Farha, Hiba Dagher, Diana Khair, Mohamad Ali Kaafarani, Zeina Jamaluddine, Patrick Cherfan

**Affiliations:** 1 *Faculty of Medicine,*; 2 *Department of Internal Medicine,*; 3 *Center for Research on Population and Health, Faculty of Health Sciences, American University of Beirut, Beirut, Lebanon. *

**Keywords:** Human Papillomavirus (HPV), Vaccination-physicians, barriers, attitudes

## Abstract

**Objectives:**

HPV infection is associated with the development of cervical and oropharyngeal cancer. HPV vaccination prevents cervical cancer, but is still not part of Lebanon’s routine vaccination schedule. As such, understanding physicians’ practice towards HPV vaccination is essential.

**Material and Methods:**

We conducted a cross-sectional study in Greater Beirut, Lebanon to assess the barriers, attitudes and clinical approach of Lebanese physicians towards HPV vaccination. We also aimed to analyze the factors associated with physicians’ barriers to HPV vaccination.

**Results:**

In total, 228 physicians completed the survey. Our results show that physicians and parents consider the cost of HPV vaccination to be a main barrier (58.9% and 80.7% respectively). Also, parents tend to have concerns about vaccine safety (78.1%), efficacy (68.6%), and lack education concerning HPV infection (81.8%). Furthermore, female physicians tend to have fewer barriers when compared to male physicians (aOR = 0.39; p-value = 0.007). Additionally, physicians who completed residency programs in the USA also showed fewer barriers when compared to physicians who completed Lebanese residency programs (aOR = 0.24; p-value = 0.040). Finally, physicians with higher knowledge score have fewer barriers when compared to those with lower knowledge scores (aOR = 0.42; p-value = 0.018).

**Conclusions:**

Physician gender, residency program and level of knowledge play a role in HPV vaccine barriers and recommendation in Lebanon. Future improvements in cost and awareness about HPV might improve vaccination rates. Creating uniform practices towards HPV vaccine is warranted to improve patient care.

## Introduction

The Human Papillomavirus (HPV) is a double-stranded non-enveloped DNA virus with multiple different serotypes already identified. Persistent HPV infection has been clearly associated with the development of several tumors, most notably cervical cancer, anogenital papilloma and oropharyngeal cancer (Walboomers et al., 1999; Clifford et al., 2003; Tolunay et al., 2014). In particular, cervical cancer is one of the leading causes of deaths for women globally, ranking as the 6th most common cancer among women aged 14 to 44 years in Lebanon (Walboomers et al., 1999; Clifford et al., 2003; Bruni L, 2017). Past research has shown that most cervical cancer cases are associated with an underlying HPV infection (Walboomers et al., 1999; Committee on Infectious, 2012).

Therefore, cervical cancer is principally preventable by the proper administration of HPV vaccine as well as appropriate screening and treatment regimens (Organization, 2014). In 2017, the World Health Organization (WHO) highlighted the importance of HPV vaccination for girls between the ages of 9 to 13, considering it as one of the 5 main goals to prevent non-communicable diseases. Furthermore, the WHO committee recommends the inclusion of HPV vaccination in national immunization programs (Organization, 2017a; Organization, 2017b). In fact, by 2018, around eighty countries worldwide introduced HPV vaccination as part of their routine vaccination schedule (Database, 2018). However, Lebanon is still not one of those countries, and hence, HPV vaccination is still not mandatory. Furthermore, previous studies have shown low rates of HPV awareness in Lebanon (Abou El-Ola et al., 2018).

For the above-mentioned reasons, HPV vaccination among Lebanese patients remains highly dependent on physicians’ recommendations (Vadaparampil et al., 2011). The following correlation makes it crucial to understand the different barriers that Lebanese physicians face regarding HPV vaccination. It is also essential to understand the attitudes of Lebanese physicians towards HPV infection and vaccination, since the following correlates with physicians’ vaccine recommendation (Vadaparampil et al., 2013).

This project is a follow up on our previous study (Abi Jaoude et al., 2018). Here, we aim to identify the barriers, attitudes and clinical approach of Lebanese physicians towards HPV vaccination. Also, we aim to analyze specific associations between physician demographics/characteristics and barriers to HPV vaccination. 

## Materials and Methods

*Study Design and Questionnaire*

A cross-sectional study was designed to assess Lebanese physicians’ barriers and attitudes regarding HPV vaccination. A survey was thus administered to physicians by telephone, e-mail or in person during the months of February and March 2017. The survey included a section on demographics adapted from Tolunay et al. (Tolunay et al., 2014). A section on knowledge related to HPV infection and vaccination was adapted from Vadaparampil et al. (Vadaparampil et al., 2011). Additionally, a section on vaccination barriers was also adapted from Tolunay et al. and Vadaparampil et al. (Vadaparampil et al., 2011; Organization, 2014). 

*Study Population*

The study included physicians practicing in Obstetrics and Gynecology, Pediatrics, Family Medicine and Infectious Diseases. The sample consisted of all physicians registered in the Lebanese Order of Physicians in January 2017 and practicing in the Greater Beirut area. We attempted to contact all listed physicians in the Lebanese Order of Physicians that met the above inclusion criteria (N=1168). Potential participants were contacted by phone to obtain informed consent for participation in the survey. When a physician did not answer, 2 additional calls were placed. 376 of the 1168 listed physicians were not reachable due to missing contact details, wrong telephone number (s), having left the country or not answering after three phone calls. When contacted, physicians were given the choice for the survey to be administered face to face, on the phone or sent via email. When the survey was sent by email, a total of 3 email reminders followed. In total, 228 physicians gave consent and provided data for the study. All the data was entered directly on tablets or through an online survey sent by email using Open Data Kit. Participating physicians were offered the choice to complete the survey either in English or in Arabic. All study protocols were approved by the Institutional Review Board of the American University of Beirut (IRB ID: FHS.HG.10. April 19, 2017).

*Statistical Analysis*

Descriptive statistics were conducted to describe the demographic characteristics of our sample along with the frequencies of physicians’ barriers. Categorical variables are presented as frequencies and corresponding percentages. Differences in categorical variables were assessed with the likelihood ratio Chi-square. Knowledge score was grouped into two categories based on median score: lower than 73 and higher than 73. 

Barriers were divided into high number of barriers for physicians who agreed on more than 5 barriers, and low number of barriers for physicians who agreed on 5 or less barriers. 

Binary logistic regression was used to calculate unadjusted odds ratio (uOR) for the association between physician’s characteristics and whether they have a high or low number of barriers. Any variable with a two-sided significance level below 0.2 was retained and included in our multivariate analysis. Adjusted odds ratios (aOR) were calculated from the multivariate analysis.

Statistical significance was set at an alpha of 5% for a two-sided p-value. All analyses were conducted using Stata Version 13. 

## Results

*Sample Characteristics*

A detailed description of the analyzed sample is present in Table 1 of the original publication (Abi Jaoude et al., 2018). In brief, a total of 228 physician of the 792 contacted completed the survey, yielding a response rate of 28.8%. The sample consisted of similar number of males and females. The majority of physicians was married, had children and was practicing in either Pediatrics or Obstetrics and Gynecology. Most physicians completed their residency program in Lebanon (45.5%). Median HPV knowledge score was 73 out of a score of 100 (73%). 

**Table 1:** Physicians and Parents Barriers to HPV Vaccination

Questions:	Physician No. (%) who agreed^1^	Physician No. (%) Who Face Parental Barriers^2^
Concerns about vaccine safety	100 (47.8)	163 (78.1)
Concerns about vaccine efficacy	109 (52.2)	143 (68.6)
Reluctance to discuss sexuality and STIs	103 (49.8)	154 (73.8)
Vaccinated teens practicing riskier sexual behaviors	96 (46.2)	113 (54.1)
Cost of the HPV vaccination	119 (58.9)	169 (80.7)
Time it takes to discuss HPV vaccination	88 (43.1)	112 (53.4)
Adding a vaccine to the vaccine to schedule^3^	77 (37.6)	-
Failure of insurance companies to cover the vaccine cost^3^	131 (63.9)	-
Difficulty ensuring that patients will complete the 3-doses^3^	75 (36.1)	-
HPV vaccination is not mandatory in Lebanon^3^	95 (45.9)	-
Lack of parent education about HPV infection^4^	-	171 (81.8)
Parent requests that HPV vaccination be deferred^4^	-	166 (79.6)
Parent believes child is not at risk for HPV infection^4^	-	164 (78.5)
Concerns about negative media related to vaccination^4^	-	155 (74.3)

- Abbreviations: STIs, Sexually Transmitted Infections; -, Numbers are physician numbers (percentage); ^1^, Physicians answered Strongly Agree or Agree; ^2^, Physicians answered Sometimes, Often or Always; ^3^, Questions were addressed only regarding physicians' barriers; ^4^, Questions were addressed only regarding parental barriers.

**Table 2:** Univariate Analysis for Physician Factors Associated with a High Number of Barriers

	% With High Number of Barriers	uOR	95% CI
Gender			
Male	52.6		
Female	29.3	0.37*	[0.21; 0.68]
Age			
25-35	40.9		
36-45	34.9	0.77	[0.27; 2.23]
46-55	36.0	0.81	[0.31; 2.14]
56-65	55.2	1.78	[0.58; 5.46]
>65	60.0	2.17	[0.57; 8.26]
Marital Status			
Currently Married	40.0		
Single	50.0	1.50	[0.56; 3.99]
Other	36.4	0.86	[0.24; 3.05]
Children			
Yes	38.5		
No	60.9	2.48*	[1.01; 6.08]
Specialty			
Pediatrics	42.9		
Obstetrics and Gynecology	35.3	0.72	[0.25; 2.13]
Family Medicine	39.1	0.86	[0.45; 1.62]
Infectious Diseases	40.0	0.89	[0.14; 5.56]
Residency Training			
Lebanon	36.8		
USA	14.3	0.29**	[0.08; 1.05]
Europe	50.8	1.77**	[0.92; 3.43]
Knowledge Score			
Lower than 73	52.3		
Higher than 73	25.3	0.31*	[0.16; 0.58]

-Abbreviations: uOR, Unadjusted Odds Ratio, 95% CI, 95% Confidence Interval; -*: p-value < 0.05; -**, p-value < 0.2

**Table 3:** Multivariate Analysis for Physician Factors Associated with Low or High Number of Barriers

	aOR	95% CI
Gender		
Male		
Female	0.39*	[0.20; 0.77]
Children		
Yes		
No	2.22	[0.79; 6.25]
Residency Training		
Lebanon		
USA	0.24*	[0.06; 0.94]
Europe	1.34	[0.65; 2.76]
Knowledge Score		
Lower than 73		
Higher than 73	0.42*	[0.21; 0.86]

- Abbreviations, aOR, Adjusted Odds Ratio; 95% CI, 95% Confidence Interval; - *, p-value < 0.05

**Figure 1:**
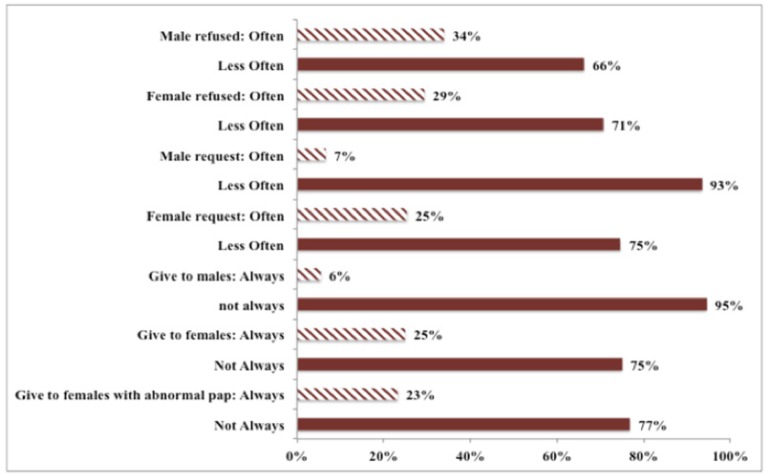
Clinical Practice of Physicians Regarding HPV Vaccination in the Last 12 Months. - The first set of questions is related to patients: Questions about male patients requesting or refusing vaccination and female patients requesting or refusing vaccination were divided into “Often” or “Less Often”. - The second set of questions is related to physicians. Questions about physicians giving the vaccine to males, females and females with abnormal pap were divided into “Always” or “Not Always”.

*Physicians and Parental Barriers to HPV Vaccination*

A set of questions was addressed to the physicians regarding what they considered barriers hindering their clinical HPV vaccine recommendation. Another set of questions addressed what parental barriers the physicians often face in clinic.

Table 1 summarizes the results of physicians and parental barriers to HPV vaccination (Table 1). Overall, parents tend to have more barriers to vaccination than physicians. The cost of the HPV vaccine appears to be a common concern for both physicians (58.9%), but even more so parents (80.7%). Also, around 64% of physicians agree that failure of insurance companies to cover the HPV vaccine cost is a barrier to HPV vaccination. On the other hand, less than half of the physicians consider that HPV vaccination not being mandatory is a barrier in itself to vaccination (45.9%).

Moreover, parents tend to have more concerns about vaccine safety and efficacy (78.1% and 68.6% respectively) when compared to physicians (47.8% and 52.2% respectively). Additionally, most parents tend to have a lack of education regarding HPV infection (81.8%) and present with concerns about negative media regarding HPV vaccination (74.3%).

Finally, parents present more barriers related to discussing sexuality and sexually transmitted infections (73.8%) when compared to physicians (49.8%). Furthermore, the majority of parents consider that their child is not at risk of HPV infection (78.5%). 

*Clinical Approach Towards HPV vaccination*

Figure 1 presents the clinical practice of physicians regarding HPV vaccination in the last 12 months (Figure 1). Around a quarter of physicians tend to always prescribe the HPV vaccine to females, regardless of pap-smear results (25% for any female, 23% for females with abnormal pap smears). On the contrary, only a minority of physicians (6%) always recommends HPV vaccination to their male patients. Additionally, female patients tend to request the HPV vaccine more often than male patients (25% vs 7% respectively). However, both female and male patients tend to refuse HPV vaccination similarly (29% of females and 34% of males). 

In their practice, approximately half of the physicians prescribed the Gardasil vaccine (46.8%), and fewer physicians prescribed the Cervarix vaccine (18.6%) or the Gardasil-9 vaccine (29.1%) (Supplementary Table 1). Lastly, most physicians consider that HPV vaccination should be part of the national vaccine schedule in Lebanon (77.5%), and that HPV vaccination prevalence would increase if insurance companies covered the vaccine cost (90.4%) (Supplementary Table 1). 

*Physician Factors Associated with a High Number of Barriers*

Physicians were divided into two categories: those having a low number of barriers (having agreed on 5 or less barrier questions (58.8%)), and those having a high number of barriers (having agreed on more than 5 barrier questions (41.2%).

Table 2 presents the binary logistic regression analyzing the association between physician’s characteristics and whether they have a high or low number of barriers (Table 2). All variables with p-values less than 0.2 were retained and included in our multivariate analysis (Table 3).

Female physicians tend to have fewer barriers when compared to male physicians with an adjusted odds ratio of 0.39 (aOR = 0.39, 95% CI = [0.20; 0.77], p-value = 0.007). Additionally, physicians who graduated from USA residency programs also showed fewer barriers when compared to physicians with Lebanese residency programs (aOR = 0.24, 95% CI = [0.06; 0.94], p-value = 0.040). Physicians graduating from European residency programs have a slightly higher number of barriers, but the following association did not show statistical significance (aOR = 1.34; p-value = 0.43). Finally, physicians with higher knowledge score have fewer barriers when compared to those with lower knowledge scores (aOR = 0.42, 95% CI = [0.21; 0.86], p-value = 0.018). 

Other factors, such as physicians’ age, marital status and specialty did not influence the physicians’ vaccination barrier status.

## Discussion

HPV vaccination is associated with a large reduction in cervical cancer rates (Organization, 2014). The following has lead the WHO to recommend HPV vaccination to girls aged 9 to 14 as a primary target population (Organization, 2017b). Vaccination is also recommended for boys and girls aged 15 or over as a secondary target (Organization, 2017b). In Lebanon, HPV vaccination is still not part of the national schedule for vaccination. Moreover, HPV vaccine is not available in all Lebanese physicians’ clinics, but can be ordered easily from most pharmacies. In this context, understanding the barriers and attitudes of Lebanese physicians towards HPV vaccination becomes essential (Vadaparampil et al., 2013). Previous studies have shown that physicians with lower barriers tend to prescribe HPV vaccination more often (Ko et al., 2010). As such, identifying and addressing physicians’ barriers to vaccination may help improve vaccination rates (Ko et al., 2010). 

We conducted a cross-sectional study in Lebanon to assess physician’s barriers, attitudes and practice regarding HPV vaccination. Our findings show that Lebanese physicians face many parental barriers that might hinder their vaccine recommendation. On the contrary, Lebanese physicians tend to have fewer barriers to HPV vaccination, with the cost of the vaccine being the main barrier to vaccination. Furthermore, female patients request the vaccine more often than males. Finally, female gender, graduating from USA residency programs and having higher knowledge scores were all associated with having fewer vaccination barriers. 

The main barriers that physicians reported were related to cost and failure of insurance companies to cover the vaccine (Table 1). Also, around 90% of physicians consider that HPV vaccination would increase if insurance companies covered the vaccine (Supplementary Table 1). The cost barrier has been reported in previous studies. In a study by Tolunay et al. 91.6% of physicians also agreed that cost reduction would lead to increased HPV vaccination (Tolunay et al., 2014). As such, efforts to reduce the cost of the vaccine and reach an optimal cost-benefit balance are required (Regan and Donovan, 2016). 

Moreover, our study shows that female physicians tend to have fewer barriers to vaccination when compared to male physicians (Table 3). A similar trend is present in the United States of America (Rosen et al., 2018). Indeed, a study by Rosen et al. in 2018 showed that in the USA, patients of female physicians are more likely to be vaccinated than patients of males physicians (Rosen et al., 2018). Efforts should be installed to drive a more uniform approach regarding HPV vaccination across both physician genders.

Additionally, physicians graduating from USA residency programs tend to have fewer barriers compared to those graduating from Lebanon. Physicians graduating from European residency programs may have slightly more barriers. The following might contrast clinical approaches of the training programs, with US programs being more liberal towards HPV vaccination. Also, physicians with higher knowledge scores regarding HPV infection and vaccination have fewer barriers (Table 3). Increasing physicians’ knowledge in that regard is of utmost importance (Abi Jaoude et al., 2018). Previous studies have shown that physician low knowledge regarding HPV is associated with lower vaccination rates (Tolunay et al., 2014; Abi Jaoude et al., 2018). Continued education and awareness among physicians regarding HPV infection and vaccination is essential to improve vaccination rates.

HPV knowledge and awareness in not only important among physicians, but is also needed among patients and parents. Our results show that physicians are often presented with parental barriers regarding vaccine safety, efficacy, and lack of information about the vaccine (Table 1). Additionally studies performed in Lebanon and neighboring countries have shown low awareness rates regarding HPV (Alsaad et al., 2012; Ortashi et al., 2013; Abou El-Ola et al., 2018; Jradi and Bawazir, 2019). Future efforts to increase patients and parents knowledge regarding HPV are warranted in the region. A study performed in 2018 by Markovic-Denic et al. showed that better knowledge towards HPV correlated with a better quality of life and lower anxiety level regarding HPV infection and pap smear results (Markovic-Denic et al., 2018). The following correlation makes it even more crucial to increase patient’s understanding of HPV infection and vaccination. One proven way to improve patient understanding of HPV infection is to incorporate tablet-based education in clinics, and this should be considered in Lebanon when feasible (Gockley et al., 2019). 

The role of media might also play a part in affecting vaccination. In our study, physicians are commonly presented with concerns about negative media regarding HPV vaccination (74.3%). The following is a major concern, as negative media has been previously shown to decrease HPV vaccination rates (Suppli et al., 2018). Additionally, the growing power of the Internet has prompted anti-vaccination supporters to use the Web to support their cause (Hussain et al., 2018). Indeed, a recently published systematic review of studies exploring vaccination media coverage showed negative media rates reaching 75% and around 81% inaccurate information (Catalan-Matamoros and Penafiel-Saiz, 2019). As such, media might be adding to the lack of education of the public regarding vaccination benefits. Efforts should be raised to enforce proper media monitoring and insure accurate information publishing. Also, future use of media to promote, rather than discourage vaccination, should be implemented (Teoh, 2019). 

Lastly, the vast majority of participants (77.5%) consider that HPV should be included in the Lebanese national vaccine schedule (Supplementary Table 1). By 2018, around eight countries worldwide introduced HPV vaccination into their routine vaccination modules, while in Lebanon HPV vaccination is still not routine (Database, 2018). Hence, including HPV vaccination in a national vaccination schedule could help increase HPV vaccination rates, and by doing so decrease HPV infection rates, as proven in previous studies (Dillner et al., 2018).

Our study has a few limitations worth noting. The main limitation of our study is its low response rate at around 29%, despite the use of multiple contacting and survey modalities. Since no identifiers on participation were collected, we could not compare factors between physicians who did or did not participate in our study. Similar low response rates have been shown among Lebanese physicians (Usta et al., 2014). The following low response rate could be explained by potential inaccuracies in the physicians’ information provided by the Lebanese Order of Physicians. It also may be explained by a slight lack of interest of Lebanese physicians in participating in research. Also, Lebanese physicians might lack the time due to heavy clinical duties. Studies have shown that monetary incentives may improve physician response rates, and might be considered in future research (Agarwal et al., 2016). Second, the population was restricted to four specialties, practicing in Greater Beirut. Thus, our results cannot be generalized to all Lebanese physicians. Despite those limitations, our study is one of the few in the area that analyzes physicians’ clinical practice towards HPV vaccination. In a context where socio-economic factors are versatile, and a proper vaccination program is still lacking, understanding HPV vaccination practice is essential to improve future quality of care.

In conclusion, this study was conducted in Lebanon, a country that still does not include HPV vaccination as part of its vaccination schedule. The WHO clearly states the importance of HPV vaccination in protecting against HPV infection (Organization, 2017a). Our results portray the multiple barriers physicians face concerning HPV vaccine, which might hinder vaccination rates. As such, creating local uniform guidelines towards HPV vaccination and increasing knowledge and awareness of HPV infection and vaccine is of utmost importance in Lebanon and the neighboring countries. Finally, since research in neighboring countries with similar cultural factors is still scarce, our results may be significant for countries in the region as well as Lebanon. Future studies analyzing HPV awareness and knowledge are still warranted.

## References

[B1] Abi Jaoude J, Khair D, Dagher H (2018). Factors associated with Human Papilloma Virus (HPV) vaccine recommendation by physicians in Lebanon, a cross-sectional study. Vaccine.

[B2] Abou El-Ola MJ, Rajab MA, Abdallah DI (2018). Low rate of human papillomavirus vaccination among schoolgirls in Lebanon: barriers to vaccination with a focus on mothers’ knowledge about available vaccines. Ther Clin Risk Manag.

[B3] Agarwal A, Raad D, Kairouz V (2016). The effect of a monetary incentive for administrative assistants on the survey response rate: a randomized controlled trial. BMC Med Res Methodol.

[B4] Alsaad MA, Shamsuddin K, Fadzil F (2012). Knowledge towards HPV infection and HPV vaccines among Syrian mothers. Asian Pac J Cancer Prev.

[B5] Bruni L B-RL, Albero G, Serrano B (2017). Human Papillomavirus and related diseases in Lebanon.

[B6] Catalan-Matamoros D, Penafiel-Saiz C (2019). How is communication of vaccines in traditional media: a systematic review. Perspect Public Health.

[B7] Clifford GM, Smith JS, Plummer M (2003). Human papillomavirus types in invasive cervical cancer worldwide: a meta-analysis. Br J Cancer.

[B8] Committee on Infectious D (2012). HPV vaccine recommendations. Pediatrics.

[B9] Database WI (2018). Map production Immunization Vaccines and Biologicals (IVB).

[B10] Dillner J, Nygard M, Munk C (2018). Decline of HPV infections in Scandinavian cervical screening populations after introduction of HPV vaccination programs. Vaccine.

[B11] Gockley AA, Pena N, Vitonis A (2019). Tablet-based patient education regarding Human Papillomavirus vaccination in colposcopy clinic. J Low Genit Tract Dis.

[B12] Hussain A, Ali S, Ahmed M (2018). The anti-vaccination movement: A regression in modern medicine. Cureus.

[B13] Jradi H, Bawazir A (2019). Knowledge, attitudes, and practices among Saudi women regarding cervical cancer, human papillomavirus (HPV) and corresponding vaccine. Vaccine.

[B14] Ko EM, Missmer S, Johnson NR (2010). Physician attitudes and practice toward human papillomavirus vaccination. J Low Genit Tract Dis.

[B15] Markovic-Denic L, Djuric O, Maksimovic N (2018). Effects of Human Papillomavirus awareness and knowledge on psychological state of women referred to cervical cancer screening. J Low Genit Tract Dis.

[B16] Organization WH (2014). Comprehensive cervical cancer control: a guide to essential practice.

[B17] Organization WH (2017a). 140th Meeting of the WHO Executive Board discusses revised menu of policy options for addressing noncommunicable diseases.

[B18] Organization WH (2017b). Summary of the WHO Position Paper on Vaccines against Human Papillomavirus (HPV).

[B19] Ortashi O, Raheel H, Shalal M (2013). Awareness and knowledge about human papillomavirus infection and vaccination among women in UAE. Asian Pac J Cancer Prev.

[B20] Regan DG, Donovan B (2016). Balancing the cost-benefit equation for cervical cancer prevention: a moving target. Lancet Public Health.

[B21] Rosen BL, Shepard A, Kahn JA (2018). US health care clinicians’ knowledge, attitudes, and practices regarding Human Papillomavirus vaccination: A qualitative systematic review. Acad Pediatr.

[B22] Suppli CH, Hansen ND, Rasmussen M (2018). Decline in HPV-vaccination uptake in Denmark - the association between HPV-related media coverage and HPV-vaccination. BMC Public Health.

[B23] Teoh D (2019). The power of social media for HPV vaccination-not fake news!. Am Soc Clin Oncol Educ Book.

[B24] Tolunay O, Celik U, Karaman SS (2014). Awareness and attitude relating to the human papilloma virus and its vaccines among pediatrics, obstetrics and gynecology specialists in Turkey. Asian Pac J Cancer Prev.

[B25] Usta J, Feder G, Antoun J (2014). Attitudes towards domestic violence in Lebanon: a qualitative study of primary care practitioners. Br J Gen Pract.

[B26] Vadaparampil ST, Kahn JA, Salmon D (2011). Missed clinical opportunities: provider recommendations for HPV vaccination for 11-12 year old girls are limited. Vaccine.

[B27] Vadaparampil ST, Murphy D, Rodriguez M (2013). Qualitative responses to a national physician survey on HPV vaccination. Vaccine.

[B28] Walboomers JM, Jacobs MV, Manos MM (1999). Human papillomavirus is a necessary cause of invasive cervical cancer worldwide. J Pathol.

